# Sequence-Specific DNA Binding by Noncovalent Peptide–Azocyclodextrin Dimer Complex as a Suitable Model for Conformational Fuzziness

**DOI:** 10.3390/molecules24132508

**Published:** 2019-07-09

**Authors:** Zulma B. Quirolo, M. Alejandra Sequeira, José C. Martins, Verónica I. Dodero

**Affiliations:** 1 Depto. Química—Universidad Nacional del Sur, Bahía Blanca 8000 Argentina; 2 NMR and Structure Analysis, Department of Organic and Macromolecular Chemistry, Ghent University, 9000 Ghent, Belgium; 3 Faculty of Chemistry, Bielefeld University, Universitätsstr. 25, 33615 Bielefeld, Germany

**Keywords:** GCN4 mimetic, peptides–DNA, E:Z photoisomerization, conformational fuzziness

## Abstract

Transcription factors are proteins lying at the endpoint of signaling pathways that control the complex process of DNA transcription. Typically, they are structurally disordered in the inactive state, but in response to an external stimulus, like a suitable ligand, they change their conformation, thereby activating DNA transcription in a spatiotemporal fashion. The observed disorder or fuzziness is functionally beneficial because it can add adaptability, versatility, and reversibility to the interaction. In this context, mimetics of the basic region of the GCN4 transcription factor (Tf) and their interaction with dsDNA sequences would be suitable models to explore the concept of conformational fuzziness experimentally. Herein, we present the first example of a system that mimics the DNA sequence-specific recognition by the GCN4 Tf through the formation of a non- covalent tetra-component complex: peptide–azoβ-CyD(dimer)–peptide–DNA. The non-covalent complex is constructed on the one hand by a 30 amino acid peptide corresponding to the basic region of GCN4 and functionalized with an adamantane moiety, and on the other hand an allosteric receptor, the azoCyDdimer, that has an azobenzene linker connecting two β-cyclodextrin units. The azoCyDdimer responds to light stimulus, existing as two photo-states: the first thermodynamically stable with an E:Z isomer ratio of 95:5 and the second obtained after irradiation with ultraviolet light, resulting in a photostationary state with a 60:40 E:Z ratio. Through electrophoretic shift assays and circular dichroism spectroscopy, we demonstrate that the E isomer is responsible for dimerization and recognition. The formation of the non-covalent tetra component complex occurs in the presence of the GCN4 cognate dsDNA sequence (′5-..ATGA cg TCAT..-3′) but not with (′5-..ATGA c TCAT..-3′) that differs in only one spacing nucleotide. Thus, we demonstrated that the tetra-component complex is formed in a specific manner that depends on the geometry of the ligand, the peptide length, and the ds DNA sequence. We hypothesized that the mechanism of interaction is sequential, and it can be described by the polymorphism model of static fuzziness. We argue that chemically modified peptides of the GCN4 Tf are suitable minimalist experimental models to investigate conformational fuzziness in protein–DNA interactions.

## 1. Introduction

### 1.1. Conformational Fuzziness and Fuzzy Complexes

Dynamics is inherently related to protein functionality and an essential process in the sophisticated transcriptional machinery. This property generates great flexibility in developing new regulatory circuits where many combinations of activators can regulate a wide variety of genes with different coactivator requirements [1,2]. Dynamics results in conformational ensembles, and indeed many proteins exist simultaneously in different yet functionally relevant conformations. The number and type of the different conformational complexes depend on the cellular environment, such as substrate gradient concentration and the different stress signals. In this sense, this conformational ‘fuzziness’ is often functionally beneficial because it can add adaptability, versatility, and reversibility to small molecule interactions, protein binding, and thereby ease of regulation not only in protein–protein interactions [2,3] but also in protein–DNA interactions. The description of biological complex systems from their ultimate constituents, i.e., atoms or molecules, is beyond our reach since they have variable patterns, whose recognition is made difficult because of their multiple features, variability, and extreme sensitivity depending on the context [3]. One chemical strategy to tackle this complexity is to obtain tailored biomolecules whose conformation can be controlled externally. Therefore, the conformational fuzziness of the biomolecular interaction can be investigated [4]. 

Finally, Tompa and Fuxreiter [1] introduced the concept of fuzzy complexes to describe binding situations in which at least one of the elements in the complex remains dynamic. Structural disorder in fuzzy complexes represents a continuum, from rather rigid polymorphic complexes displaying static disorder with only a few alternative conformations to highly dynamic random complexes [1,2,5].

Since up to now, the fundamental understanding of the spatiotemporal control of the interaction between transcription factors (Tfs) and DNA remains elusive, we envisaged designing a suitable Tf biomimetic system combining molecular recognition and supramolecular chemistry to exert the external control of the interaction. We hypothesized that a non-covalent interaction between the Tf and the DNA would be an optimal system to mimic the biological interaction. Under this circumstance, different possible complexes and their respective conformers would exist in equilibrium in the unbound and bound states. Therefore, we anticipate that the concepts of conformational fuzziness and fuzzy complexes would facilitate the analysis of our experimental model. We think that such a system and its analysis would offer a unique strategy to understand the molecular triggers of transcription and the underlying fuzzy mechanism of the Tf–DNA interaction.

### 1.2. Design of the Non-Covalent Externally Controlled Biomimetic System

**Figure 1:**
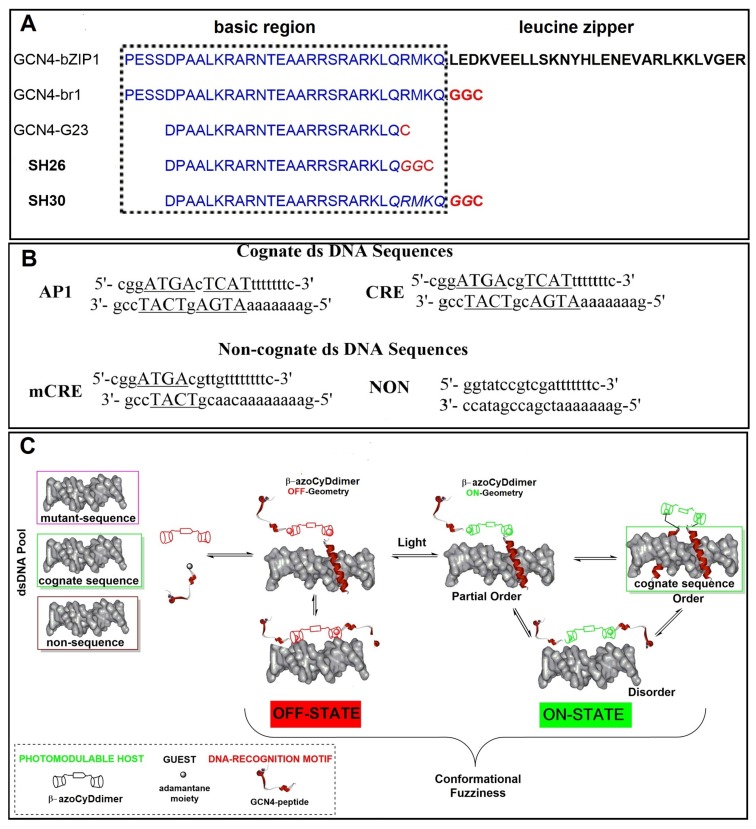
(**A**) Natural GCN4 sequence (GCN4-bzip), and different truncated GCN4 basic regions: GCN4-br1 [8], GCN4-G23 [9], SH26 [11], and SH30. (**B**) Cognate and non-cognate oligonucleotides employed in this work; the recognition and binding sequences are underlined. (**C**) Schematic representation of the possible conformational fuzziness product of the interaction among (β-azoCyDdimer)–peptide–Ad and different dsDNA. By controlling the geometry of the host–guest interaction triggered by light, it would be possible to promote dimerization and the recognition of the cognate dsDNA sequence. The structural transitions of the dimerization and recognition regions upon different partner interactions generate a structural and dynamical continuum towards the formation of the specific non-covalent tetra-component complex *only* in the case of the cognate dsDNA. Although other complexes with different stoichiometry can be formed, they are not shown to simplify the scheme.

In *Saccharomyces cerevisiae*, GCN4 transcription factor (Tf) is part of the “general control” system of amino acid biosynthesis, a network of at least 12 different biosynthetic pathways [6]. GCN4 is a member of the bZIP (basic leucine zipper) family of transcriptional activators that bind to the major groove of double-stranded DNA as a homodimer (Figure 1A) [7,8]. The natural dsDNA specific target sequences of the GCN4 dimers are the activator protein 1 binding site (AP1, 5′-..ATGAcTCAT..-3′) and the related cyclic AMP response element (CRE, 5′-..ATGAcgTCAT..-3′). GCN4 is composed of a C-terminal leucine zipper sequence that associates into non-covalent, parallel, alpha-helical dimers, and an N-terminal basic region necessary for binding DNA. The basic regions are disordered in the absence of DNA and form alpha helices only when as a homodimer binds to the cognate DNA (Figure 1A). Therefore, GCN4 Tf is a suitable protein model to investigate the relationship between structural order/disorder by binding with its mediators or cofactors [9] and in the context of protein–DNA interactions. 

Pioneering work in the construction of GCN4 mimetics was performed by Kim et al. [10]. They showed that the replacement of the leucine zipper segment of GCN4 by a cysteine capable of dimerization through disulfide bond formation (GCN4-br1, Figure 1A), allowed specific recognition of its consensus DNA sequences, CRE (′5-..ATGA cg TCAT..-3′) and AP1 (′5-..ATGA c TCAT..-3′), with a nanomolar affinity at 4 °C (Figure 1B). Importantly, the monomeric 34-amino acid sequence was not capable of binding to dsDNA by itself, pointing to dimerization as an essential prerequisite. Furthermore, it was possible to trim the basic region to only 23 amino acids, while maintaining the specific recognition of consensus sequences by the covalent cysteine dimer at affinities around 10 nM at 4 °C [11]. In an alternative approach, Morii et al. [12] used a 23-residue peptide to form a non-covalent heterodimeric complex through host–guest supramolecular interactions. One monomer was equipped with an adamantane group (Ad) and the other with a β-cyclodextrin group (β-CyD). In the presence of the cognate dsDNA, they formed a non-covalent heterodimer. The heterodimer obtained by formation of the inclusion complex between the β-CyD (host) and the Ad (guest) was capable of selectively recognizing the CRE binding domain [12]. To address and control the reversibility of the binding between the GCN4 Tf mimetic and its cognate dsDNA, Mascareñas’ group employed an azobenzene moiety covalently attached to the basic region of GCN4 with 26 amino acids [13]. This covalent system was capable of selective binding and recognizing its target dsDNA after UV-light irradiation when the azobenzene was in the (Z) conformation, effectively creating an off–on system. More recently, the same group designed a stimuli-responsive system that targeted different dsDNA triggered by different metals [14]. Building on these previous examples and to address the fuzziness of the Tf–DNA interaction, we envisaged employing an alternative homodimeric non-covalent strategy where the addition of an external molecule would promote not only dimerization but also control over the specific DNA recognition by the geometry of host–guest interactions. In this hypothetical system, the cognate protein–DNA interaction would build a non-covalent tetra-component complex only if the geometry of the host–guest interaction would promote peptide dimerization and a concomitant specific DNA binding occurs. However, many different complexes and their conformers are possible. Considering the non-covalent approach, there are opportunities to control externally the system and to modulate the number and type of complexes and their conformers depending on the geometry of ligand and the dsDNA sequence (Figure 1C).

To address host–guest interactions and switchability of the dimerization, we employed a molecule with two β-cyclodextrin units connected via an azobenzene group, a β-CyD-azobenzene–β-CyD (β-azoCyDdimer). This double host would be capable of binding two basic regions of GCN4 if the peptide were equipped with the adamantane (Ad) guest moiety forming a conjugated peptide–Ad molecule.

In general, double-host β-CyDs bind with higher affinity to Ad than simple β-CyD because of a cooperative effect, as described by Breslow et al. [15]. Moreover, the strategy of a non-covalent β-CyD double host brings the possibility to modulate the geometry and inclusion properties of the peptide–(β-azoCyDdimer) interaction by the photoisomerization of the azobenzene moiety. Thus, the photoswitchable host will work as an allosteric receptor in the petide-DNA interaction. The adamantane moiety was incorporated into two minimal mimetics of GCN4 containing 26 amino acid and a second one with 30 amino acids, SH26 and SH30, respectively (Figure 1A). Both sequences contain a flexible GG linker, and the longer SH30 sequence contains the sequence RMKQ in the C-terminus that should enhance the binding capacity to the cognate dsDNA sequences [9]. To investigate the recognition capacity and the sequence specificity of the peptide–azoβ-CyD(dimer)–peptide–DNA interaction, electrophoretic mobility shift assays (EMSA), and circular dichroism spectroscopy (CD) in the presence of the cognate and non-cognate sequences were tested (Figure 1B). From EMSA experiments the stoichiometry of the interaction, if any, could be identified; meanwhile, the CD would provide us information about the transition from disorder-to-partial-order or disorder-to-order in the bound state by monitoring the alpha helix content of the peptide.

In summary, we hypothesized the formation of a non-covalent tetra-component complex, (peptide–azoβ-CyD(dimer)–peptide–DNA), only if the required geometry to drive dimerization and recognition is provided by the host–guest interaction, which depends ultimately on light illumination (Figure 1C). In this way, new possibilities may arise to investigate the different conformations that are involved in DNA recognition triggered by an external stimulus and evaluate the concept of fuzziness in protein–DNA interactions.

## 2. Results

### 2.1. Synthesis and Characterization of the Allosteric Receptor and Adamantyl Substituted Peptides

#### 2.1.1. Allosteric Receptor Synthesis and Characterization

**Figure 2:**
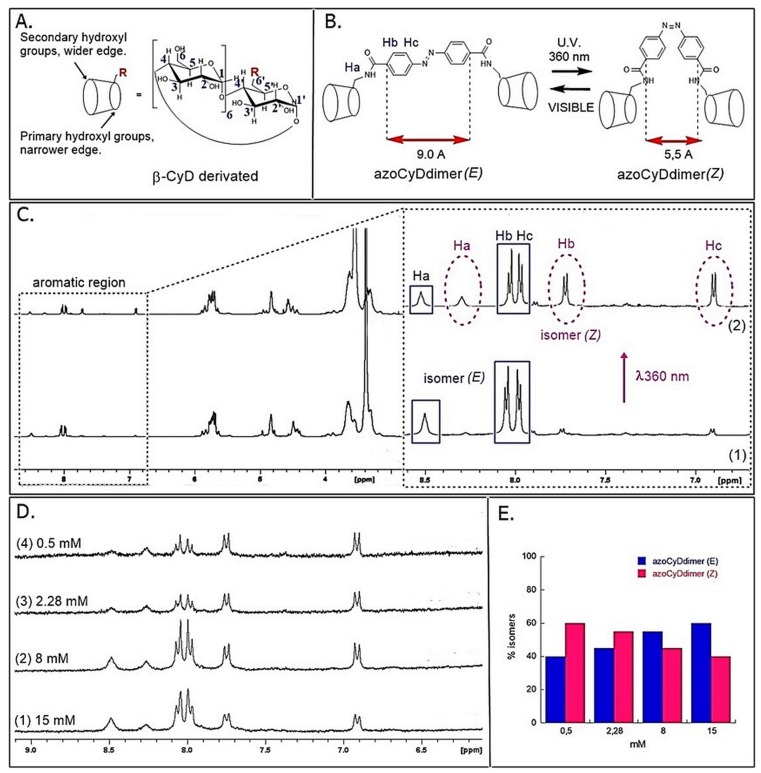
(**A**) Structure of the allosteric receptor. (**B**) Representation of the photoisomerization of the azoCyDdimer. (**C1**) ^1^H-NMR-500MHz spectra of azoCyDdimer (15 mM) at 25 °C (DMSO-*d*_6_). (**C2**) ^1^H NMR spectra of azoCyDdimer after 4 h irradiation with UV light (λ = 360 nm). Isomer (E) signals are marked with full lines and isomer (Z) signals with dotted lines. (**D**) Independent ^1^H-NMR (300MHz) experiments of the photoisomerization of azoCyDdimer at different initial concentrations, showing the aromatic region after 20 min of irradiation at 360 nm in DMSO-d6, 25 °C (see Materials and Methods). (**E**) Bar graph of E:Z isomer ratio obtained by integration of the ^1^HNMR signals in D.

Herein, we simplified the synthesis of the β-CyD double host, azoCyDdimer [16] (Figure 2A) by direct coupling of 4,4′-bis (carboxy) azobenzene acid and mono-6-amino-6-deoxy-β-cyclodextrin (β-CyDNH_2_) in DMF using HATU, as a coupling agent and DIPEA. The reaction was monitored by TLC until the disappearance of β-CyDNH_2,_ and purified by preparative HPLC obtaining 20% yield. In general, monosubstituted cyclodextrins (CyD) lack the high symmetry of free or symmetrically substituted CyD and, therefore, present substantially more complex NMR spectra. Previously, the azoCyDdimer was not entirely characterized [14]. Taking this into account, we present here the complete characterization of the azoCyDdimer by a combination of 2D NMR experiments. The azoCyDdimer structure, made by two CyD linked through an azobenzene unit, was initially studied by ^1^H NMR in DMSO (Figure 2A,C, and Supplementary Materials, Figure S1). Two distinct anomeric proton signals at δ = 4.96 ppm for H-1 and δ = 4.83 ppm for H-1′ were observed (with a relative area of 12:2), corresponding to the pattern of monosubstituted CyD. The –OH2 and –OH3 groups were observed between 5.70 to 5.82 ppm. The other group of signals between 3.20 and 3.80 ppm corresponded to the rest of the protons of the glucose units and suffered from substantial overlap (see Supplementary Materials, Figure S1). Nevertheless, the complete assignment could be obtained through the use of COSY, TOCSY, ^1^H-{^13^C} HSQC, and HMBC.

Regarding the signals of the photomodulable connector, two doublets of 4H area were observed at δ = 7.98 (*J* = 8.6 Hz) and δ = 8.04 (*J* = 8.6 Hz), which corresponded to a substituted azobenzene derivative in position 4.4 ‘ (isomer (E)). Using a multiplicity edited HSQC experiment, CH units were differentiated from CH_2_, while the absence of correlations allowed the identification of signals corresponding to the NH and OH groups. The analysis was completed by HMBC, which allowed establishment of longer distance correlations between ^1^H and ^13^C over two or three bonds. This enabled the correlation of Hb at 8.00 ppm (doublet) with the carbonyl of the amide, while a weak correlation was also observed between Cb and the H of the amide thus confirming its integrity (see Supplementary Materials, Figure S2 and S3). 

Furthermore, we evaluated the E:Z photoisomerization of the dimer using ^1^H NMR and UV–Vis spectroscopy (Figure 2C and see Supplementary Materials, Figure S4). Changes in the E:Z ratio after photoisomerization were calculated from the integration of the corresponding areas of the azobenzene protons, as shown in Figure 2C. The signals corresponding to the Z isomer appeared at the higher field than the corresponding signals of the E isomer due to the different contribution of the diamagnetic anisotropy of both benzene rings in the E:Z isomers. As mentioned, the ^1^H-NMR spectrum of the E isomer (Figure 2(C1)) had two doublets at 8.03 ppm (*J* = 8.6 Hz) and 7.97 ppm (*J* = 8.6 Hz) corresponding to the aromatic protons Hc and Hb of the azobenzene linker group, and a singlet at 8.52 ppm corresponding to the protons of the amide group. After the isomerization, a decrease of these signals was observed while three new signals corresponding to the Z isomer appeared: two doublets at 7.72 ppm (*J* = 8.4 Hz) and 6.90 ppm (*J* = 8.4 Hz) and a singlet at 8.20 of the H corresponding to the amide bond (Figure 2(C2)).

Previously, it was reported that azoCyDdimer forms supramolecular aggregates in water above the concentration 1 mM, while below this concentration it reaches the photostationary state of E:Z 60:40 as determined by UV–Vis spectroscopy [16]. Here, we analyzed the dependence of the concentration and isomerization by the integration of the peaks using ^1^H-NMR in DMSO. We observed that the most diluted samples favored the formation of the Z isomer, from E:Z ratios of 40:60 (0.5 mM) to 60:40 (15 mM) (Figure 2D,E). 

#### 2.1.2. Peptide Derivatives Synthesis

**Figure 3:**
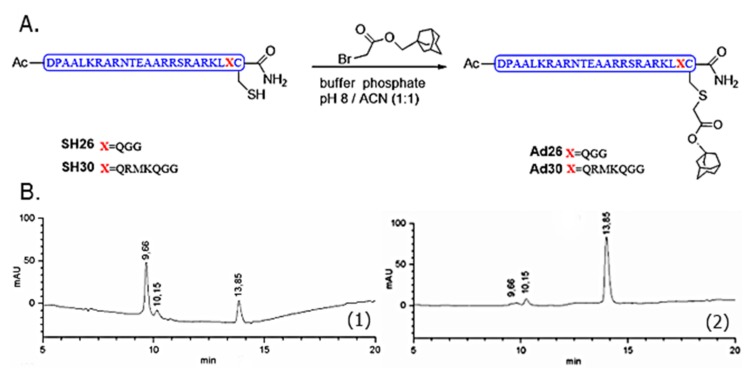
(**A**) GNC4 peptides derivatives. (**B**) RP-LC–MS-monitoring of the reaction between SH30 and bromoacetyl adamantane to obtain Ad30 at 5 (1) and 120 (2) min.

To obtain a minimal GCN4 mimick, we synthesized two adamantane GNC4 derivatives (Ad26 and Ad30, Figure 3A). Both peptides were obtained by coupling the bromoacetyladamantane with the thiol group of Cys26 or Cys30, respectively.

We took advantage of the nucleophilic reactivity offered by the thiol group of cysteine in a solution using the completely deprotected peptide. The synthesis of the new Ad30 was performed following the established protocol for Ad26 [17,18]. In Figure 3B, LC–MS chromatogram shows the evolution of the reaction, which was verified by ESI. After 2 h of reaction, the peak of SH30 (tr 9.56 min) disappeared and a new peak at 13.85 min appeared, whose m/z corresponded to the final product Ad30.

Both Ad26 and Ad30 peptides were employed as the minimal GCN4 derivatives to evaluate the interaction strategy of non-covalent homodimers. Furthermore, we employed the corresponding disulfur dimers SS52 and SS60 as positive controls of the peptide–DNA interaction [10,17,18].

### 2.2. Peptide–dsDNA Interaction

#### 2.2.1. Electrophoretic Mobility Shift Assays (EMSA)

**Figure 4:**
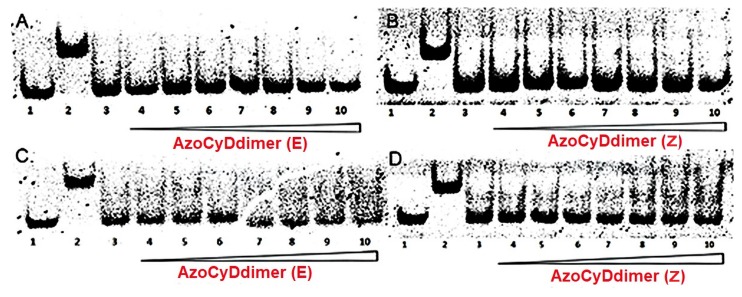
EMSA analysis of DNA binding of Ad26 peptide in the presence and absence of azoCyDdimer. (**A**) Lanes 1–10: 50 nM AP1 (′5-cggATGA c TCATtttttttc-3′); lane 2: 300 nM SS52; lanes 3–10: 200 nM Ad26 and 0, 0.5, 1, 2, 5, 20, 50, 100 equivalents azoCyDdimer (E). (**B**) Lanes 1–10: 50 nM AP1 (′5-cggATGA c TCATtttttttc-3′); lane 2: 300 nM SS52; lanes 3–10: 200 nM Ad26 and 0, 0.5, 1, 2, 5, 20, 50, 100 equivalents azoCyDdimer (Z). (**C**) Lanes 1–10: 50 nM CRE (′5-cggATGA cg TCATttttttt-3′); lane 2: 300 nM SS52; lanes 3–10: 200 nM Ad26 and 0, 0.5, 1, 2, 5, 20, 50, 100 equivalents azoCyDdimer (E). (**D**) Lanes 1–10: 50 nM CRE (′5-cggATGA cg TCATtttttttc-3′); lane 2: 300 nM SS52; lanes 3–10: 200 nM Ad26 and 0, 0.5, 1, 2, 5, 20, 50, 100 equivalents azoCyDdimer (Z). DNA was detected by fluorescent staining with SYBR-gold.

When a GCN4 peptide binds to a cognate dsDNA, it induces a delay in the migration of the peptide–dsDNA complex in comparison with free dsDNA when using EMSA. This interaction leads to a characteristic shift in the position of the associated band, leading to the observation of two bands with an intensity ratio reflecting the equilibrium involved in complex formation. EMSA experiments require a minimal amount of sample in the nM range, making it an ideal and common technique to obtain qualitative information of the respective peptide–DNA interaction [10,11,12,13,17,18,19]. The DNA-binding properties of the synthetic peptides were studied by EMSA under non-denaturing conditions and using SYBR-gold for DNA staining. Initially, the incubation of Ad26 in the presence or absence of azoCyDdimer to both AP1 and CRE dsDNA (Figure 1B) showed no interaction bands, as shown in Figure 4. In this case, a new band could only be observed for the positive control SS52, which is the SH26 disulfide dimer (Figure 4, lane 2), confirming the negative result. It was noticeable that the interaction of Ad26 with CRE (Figure 4C,D) produced a decrease in the free DNA band. We hypothesized that this behavior might be a consequence of the formation of different types of complexes with the dsDNA, which would make diffuse broadband under both conditions. Nevertheless, no strong interaction was detected at the nM range. The azobenzene molecule extends over a considerably different distance when considering the E and Z isomers, being theoretically about 9 to 5.5 Å, respectively [20,21,22]. When we tried to mimic the binding system of GCN4, this distance may not have been adequate for the specific interaction with the short sequence and its putative dsDNA binding site. Considering that no new migration band was observed in the nM, this system was not suitable for further evaluation, mainly because upon increasing peptide concentration the unspecific interactions started to be relevant.

**Figure 5:**
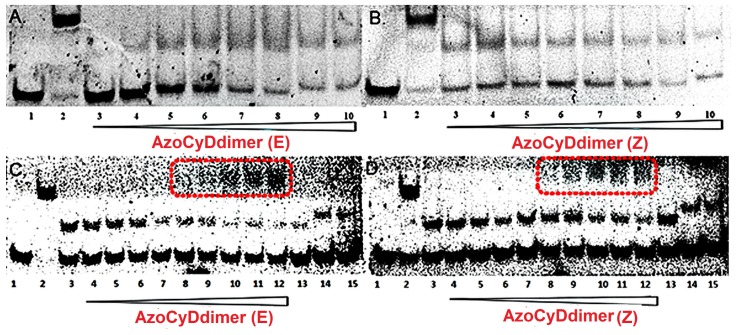
EMSA analysis of DNA binding of Ad30 peptide in the presence and absence of azoCyDdimer. (**A**) Lanes 1–10: 50 nM AP1 (′5-cggATGA c TCATtttttttc-3′); lane 2: 300 nM SS60; lanes 3–10: 200 nM Ad30 and 0, 0.5, 1, 2, 5, 20, 50, 100 equivalents azoCyDdimer (E). (**B**) Lanes 1–10: 50 nM AP1 (′5-cggATGA c TCATtttttttc-3′); lane 2: 300 nM SS60; lanes 3–10: 200 nM Ad30 and 0, 0.5, 1, 2, 5, 20, 50, 100 equivalents azoCyDdimer (Z). (**C**) Ad30 and azoCyDdimer (E): lane 1–13: 50 nM CRE (′5-cggATGA cg TCATtttttttc-3′); lane 2: 100 nM SS60; lanes 3–15: 200 nM Ad30; lanes 3–10: azoCyDdimer (E): 0, 0.5, 1, 2, 10, 50, 100, 500 eq.; lane 11–12: excess of azoCyDdimer (E) (0.15 mM); lane 13: Ad30 200 nM; lane 14: 50 nM ds mCRE (′5-cggATGAcgttgtttttttc-3′): 200 nM Ad30; lane 15: 50 nM mCRE (′5-cggATGAcgttgtttttttc-3′): 200 nM Ad30, 0,5 eq azoCyDdimer(E). (**D**) Ad30 and azoCyDdimer (Z) lane 1–13: 50 nM CRE (′5-cggATGA cg TCATtttttttc-3′); lane 2: 100 nM SS60; lane 3–10: 200 nM Ad30 and azoCyDdimer (Z): 0, 0.5, 1, 2, 10, 50, 100, 500 eq.; lane 11–12: Ad30: 200 nM and excess of azoCyDdimer (Z) (0.15 mM); lane 13: Ad30 200 nM; lane 14: 50 nM ds mCRE (′5-cggATGAcgttgtttttttc-3′); 200 nM Ad30; lane 15: 50 nM ds mCRE (′5-cggATGAcgttgtttttttc-3′): 200 nM Ad30, 0,5 eq. azoCyDdimer (Z). DNA was detected by fluorescent staining with SYBR-gold.

On the other hand, incubation of the derivative Ad30 with AP1 showed a new migration band, the molecular weight of which corresponded to the Ad30 monomer that binds to dsDNA (Figure 5A,B, lane 2, compare with the positive interaction with the covalent dimer of SH30, named as SS60) [12,17]. It seems that the monomer Ad30 interacted with one half of the recognition site, but no tri or tetra-component complex was obtained, leading us to conclude that this interaction should be considered as unspecific, although with high affinity. Interestingly, once the azoCyDdimer was present in the incubation mixture with Ad30 and the CRE sequence, two bands appeared, one corresponding to the above-mentioned unspecific interaction, and the second one with a retardation band compatible with a higher order complex (Figure 5C,D). By increasing the concentration of azoCyDdimer, the formation of the second complex was favored. This new migration band was compatible with the size of the tetra-component system Ad30–AzoCyDdimer–Ad30–CRE that migrated less than the disulfide dimer (compare with lane 2, Figure 5). The intense band of specific binding was only observed in the presence of an excess of azoCyDdimer enriched in the isomer (E), to the detriment of the nonspecific binding that nearly disappeared at the highest azoCyDdimer concentration (Figure 5C, lanes 10–12). A similar result was observed when the azoCyDdimer (Z) was added to the incubation mixture; however, in this case, the specific band was more diffuse, showing the existence of different complexes in the mixture (Figure 5D, lanes 9–12).

Additionally, in the presence of the azoCyDdimer (Z), the relative intensity of the non-specific band did not show a significant decrease in comparison with the specific one. To further investigate the specificity of the interaction, we employed a dsDNA with half CRE sequence (mCRE), (Figure 5, in lanes 14 and 15, gels C and D) showing the migration of the band compatible with a complex monomer Ad30–azoCyDdimer–mCRE. The slightly different migration profile could be a different migration of both dsDNA sequences. It is important to mention that under this circumstance, it seems that the mixture enriched in E isomer (95:5) could form the cooperative complex. Therefore, the mixture enriched in the Z isomer, which still contained 60% of E isomer after photo-illumination, could also bind.

Considering the positive binding of the Ad30–azoCyDdimer–Ad30 system and the CRE sequence, we further evaluated its interaction by circular dichroism (CD) spectroscopy.

#### 2.2.2. Circular Dichroism Spectroscopy

**Figure 6:**
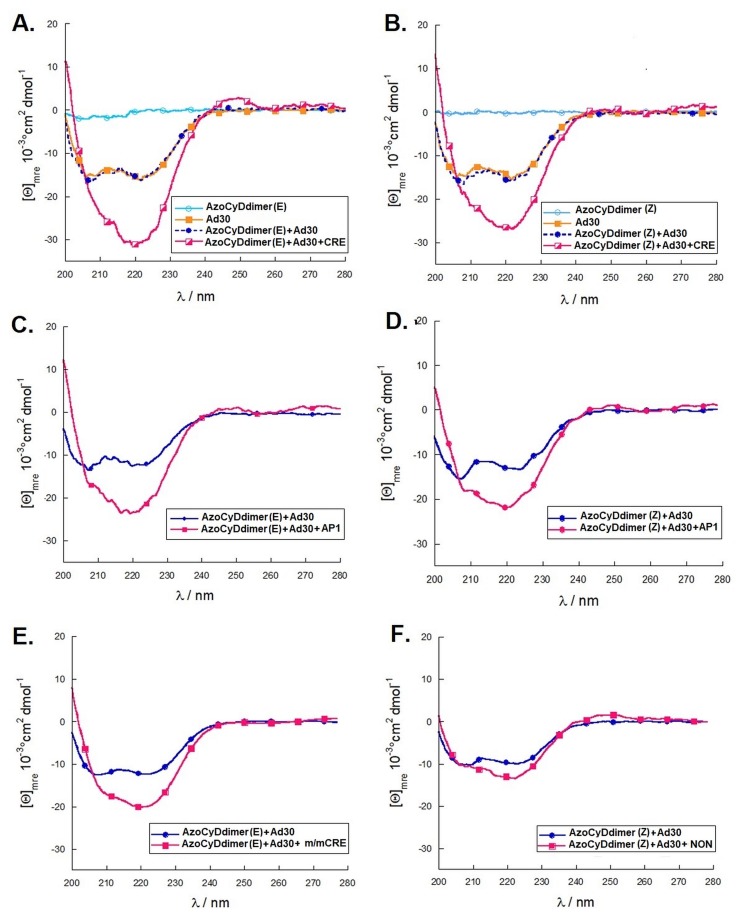
Circular dichroism evaluation of the interaction of Ad30 (**A**) with azoCyDdimer (E) and its cognate sequence CRE (′5-cggATGA cg TCATtttttttc-3′), (**B**) with azoCyDdimer (Z) and its cognate sequence CRE (′5-cggATGA cg TCATtttttttc-3′), (**C**) with azoCyDdimer (E) and its cognate sequence AP1 (′5-cggATGA c TCATtttttttc-3′), (**D**) with azoCyDdimer (Z) and its cognate sequence AP1 (′5-cggATGA c TCATtttttttc-3′), (**E**) with azoCyDdimer (E) and mCRE (′5-cggATGAcgttgtttttttc-3′), and (**F**) with azoCyDdimer (E) in the presence of a random sequence, NON (′5-ggtatgcgtcgatttttttc-3′). In all experiments, the band of dsDNA was subtracted; for further experimental details refer to Materials and Methods section.

There is a proportional relationship between the amount of α-helix and the intensity of the negative signal at 222 nm in the circular dichroism spectrum, especially useful for study the bZIP-DNA interactions [23,24]. In the case of GCN4, which is disordered in the absence of its cognate dsDNA, there is an increase in its folding from random coil to α-helix when it interacts specifically with its consensus DNA. Briefly, Ad30 solution was added to a 5 µM solution of azoCyDdimer (E) in phosphate buffer to monitor any change due to the interaction. A decrease of the band at 222 nm from −0.2118 (CyDdimer only) to −16.201 °cm^2^dmol^−1^ was observed when Ad30 was added (Figure 6A). In the presence of the CRE sequence (′5-..ATGA cg TCAT..-3′), a decrease of the same band was observed because of the interaction with the CRE binding site at −30,666 °cm^2^dmol^−1^, validating the specific interaction of the Ad30–azoCyDdimer (E)–Ad30 system and CRE obtained by EMSA assays (Figure 5C). The sample was irradiated with UV light (360 nm) to increase the ratio of isomer Z in the photostationary state, and a slight change in the band at 222 nm was observed, from −29.968 °cm^2^dmol^−1^ at 20 min to −28.953 °cm^2^dmol^−1^ at 40 min. As in the case of isomer (E), the solution enriched with azoCyDdimer (Z) did not show a significant contribution of the ellipticity at 222 nm (−0.2956 °cm^2^dmol^−1^) (Figure 6B). With the addition of the solution of Ad30 peptide, the value of the band at 222 nm decreased to −15.797 °cm^2^dmol^−1^ and then when CRE was added, the second decrease in ellipticity was observed, reaching the value of −26.709 °cm^2^dmol^−1^, which showed lower alpha-helical content in comparison with the azoCyDdimer (E). Once this mixture was irradiated with white light, the signal at 222 nm decreased slightly to −27.034 °cm^2^dmol^−1^.

On the other hand, in the interaction of both isomers with AP1, (′5-..ATGA c TCAT..-3′), the band at 222 nm decreased to −22.974 °cm^2^dmol^−1^ in the case of the isomer E and to −21.408 °cm^2^dmol^−1^ when the isomer Z was present (Figure 6C,D). Both results were in the same direction as the EMSA, showing that the geometry of the dimer was not favorable for the interaction with AP1, which had only one base spacer between both recognition sites.

Finally, we tested the unspecific interaction with the half sequence of mCRE (′5-..ATGA cg..-3′), detecting a decrease of the molar ellipticity to −20.214 °cm^2^dmol^−1^. This value, as expected, was lower than the one observed with the full sequence (compare Figure 6A,E). The observed decrease in molar ellipticity could be due to the insertion of the Ad30 monomer in the major groove and interaction with the bases of the half sequence (′5-..ATGA cg..-3′). To investigate the role of the phosphates of the DNA and their binding to the basic Ad30, we experimented with a random DNA sequence that does not possess a cognate binding site, named NON (′5-ggtatgcgtcgatttttttc -3′) (Figure 6F). For this case, a slight decrease in the signal from −10.389 to −14.323 °cm^2^dmol^−1^ was observed. From that experiment, we can exclude that the unspecific interaction with the negative phosphates of DNA and the basic GCN4 peptide has a role in the interaction of Ad30.

These results are in agreement with those obtained by EMSA, showing that Ad30 in the presence of azoCyDdimer in the E configuration presented a specific binding to CRE sequence forming the tetra-component complex; meanwhile, in the Z configuration, the helical content was lower. Considering that the E isomer is an equilibrated mixture 95:5 (E:Z) and the Z isomer is a 60:40 (E:Z) mixture, we hypothesized that the E isomer has the right geometry to form the specific tetra-component complex. The CD signal of the interaction with AP1 was in the same range of those observed for the half-recognition sequence, mCRE. This shows the feasibility of a monomer Ad30–AzoCyDdimer to bind to the sequence (′5-..ATGAcg..-3′), but not as a dimer as detected by the EMSA experiment (Figure 5A,B). Importantly both CD and EMSA experiments demonstrated the feasibility of formation of the non-covalent complex Ad30–AzoCyDdimer (E)–Ad30 that interacts preferably with CRE sequence, (′5-..ATGA cg TCAT..-3′). 

#### 2.2.3. Competitive Binding Assay with 1-Adamantane Acetic Acid by CD and EMSA Experiments

**Figure 7:**
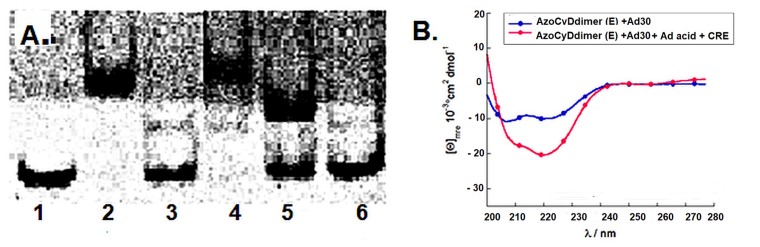
(**A**) EMSA analysis of DNA binding of Ad30–azoCyDdimer (E)–Ad30 in the absence and presence of 1-adamantane acetic acid. Lanes 1–5: 50 nM CRE (′5-cggATGA cg TCATtttttttc-3′); lane 2: 300 nM SS60; lane 3: 200 nM Ad30; lane 4: 200 nM Ad30 and 100 equiv azoCyDdimer (E); lane 5: 200 nM Ad30, 100 equiv azoCyDdimer (E) and 200 equiv 1-adamantane acetic acid; lane 6: 50 nM NON, 200 nM Ad30, 100 equiv of azoCyDdimer (E) and 200 equiv 1-adamantane acetic acid. (**B**) CD spectra of the interaction Ad30–AzoCyDdimer (E)–Ad30–CRE in presence and absence of 1-adamantane acetic acid.

To further corroborate the formation of the inclusion complex Ad–βCyD in the system Ad30–AzoCyDdimer (E)–Ad30, we hypothesized that such interaction would collapse upon addition of an excess amount of a competitive β-cyclodextrin binder, such as 1-adamantane acetic acid [12]. Under this circumstance, the helical content would decrease due to the dimer collapse, and the specific band in the EMSA experiment should disappear. Following this hypothesis, we evaluated first the system by the EMSA experiment (Figure 7A). 

It was observed that the tetra-component complex (lane 4) in the presence of an excess of the 1-adamantane acetic acid was not formed (lane 5); instead, only the unspecific shift band of the complex peptide:DNA (1:1) was detected. In the case of the interaction of the NON, any shift band was detected in the presence of the competitive β-cyclodextrin binder (lane 6). By CD, it was observed that in the presence of an excess of 1-adamantane acetic acid, the negative band at 222 nm reached only the value of −20.409 °cm^2^dmol^−1^ instead of the expected −30,666 °cm^2^dmol^−1^ for the dimer (Figure 7B). The obtained value was in the range of the unspecific interaction obtained by interaction with the half-sequence mCRE sequence (−20.214 °cm^2^dmol^−1^) (see Figure 6E). These results confirm the relevance of the βCyD–Ad host–guest interaction to promote dimerization and thus trigger the specific interaction with the target dsDNA.

## 3. Discussion

Transcription factors (Tfs) are useful proteins to evaluate the concept of fuzziness in protein–protein and protein–DNA interactions [1,2,7,25,26,27,28,29]. For GCN4 Tf, it has been hypothesized that the binding to DNA occurs sequentially in such a way that a monomer is first assembled into the major groove of DNA, followed by dimerization with a second GCN4 Tf unit [30]. Generally, mimetics of the GCN4 Tf possess a high degree of randomness, but in the presence of a dimerization motif and their cognate ds DNA, there is a substantial increase of α helical structure due to structure inducing complex formation as a result of the specific binding [9,10,11,12,13,14]. Conformational dynamism and heterogeneity enable context-specific functions to emerge in response to changing environmental conditions and, furthermore, allow a single structural motif to be used in multiple settings [14]. The sequential interaction pathway is a natural strategy that prevents dimers from being trapped for a relatively long time in non-consensus sequences. In the case of GCN4 mimetics, the dimerization motif (Figure 1A), which is not involved in the DNA binding interface, is generally conformationally unaffected by binding to the DNA. Thereby, it retains the capability to modulate the interaction [31]. In general, heterogeneous conformational segments can increase binding affinity. This conformational flexibility and heterogeneity of proteins represent their fuzziness [1,2,3,4,5,6]. The classical framework of protein interactions establishes that there is a deterministic relationship between protein sequence and function. Based on this, a distinguished three-dimensional arrangement of the amino acids is a prerequisite for a given biological activity and is unambiguously encoded in the sequence [32]. However, protein functions are modulated by different mechanism triggered by different effectors. The effector perturbs one site and thereby leads to altered activity in a second, substrate site [33]. In our system, the effector was the azoCyDdimer, which triggered host–guest interaction of β-CyD of the dimer and two GCN4 peptides containing each an adamantane moiety. The different geometry the azoCyDdimer was previously calculated as 9.1 Å and 6.6 Å for E and Z isomers, respectively [16]. As confirmed here, the azoCyDdimer exists under two photo-states: the first thermodynamically stable with an E:Z isomer ratio of 95:5 and the second obtained after irradiating with ultraviolet light, E:Z 60:40 ratio. Taking into account, the large geometrical difference between both isomers and the relevance of this distance to form a suitable complex, we had hypothesized that it would be unlikely that both isomers could form a homodimer leading to the same specific dsDNA interaction. Considering that the first and second photo-stationary state contains a major proportion of E isomer 95% and 60%, respectively, it can be anticipated that if the E isomer is the responsible of the tetra-component complex, the specific interaction would be observed under both illumination conditions. However, some differences in binding affinity can be expected. On the contrary, if the Z isomer would give the right geometry for the peptide–DNA interaction, the positive interaction would be observed only mainly in the second photostationary stage where the Z isomer is present in a significant concentration (40%). Nevertheless, if both isomers would contribute equally to the tetra-component complex, similar behavior in all the experiments would be expected.

We performed two complementary sets of experiments, EMSA and CD, to evaluate the system. As aforementioned, from EMSA experiments the stoichiometry of the interaction, if any, was identified. Meanwhile, the CD gave information about the transition from disorder-to-order and disorder-to-partial-order in the bound state. In such a case, the value of the molar helicity per residue would let us set a scale of the order obtained for each interaction and connecting them with the stoichiometry obtained in the EMSA experiment. Considering the theory of fuzzy complexes [1], the disordered-binding elements of GCN4 mimetic may undergo three types of structural transitions upon interaction with the target dsDNA. First, let us consider the disorder-to-order transition to adopt a stable, well-defined conformation in the bound state [32]. This is also referred to as coupled folding to binding [34]. Second, upon partner recognition, a transition from disorder-to-partial order could take place in shallow, often hydrophobic binding pockets. In this case, the interface was generated by many redundant contacts and few specificities, as observed by the formation of the complex DNA–Ad30 and DNA–Ad30–azoCyDdimer (E) [32]. Third, a disorder-to-disorder transition may occur upon binding of the Ad30–azoCyDdimer–Ad30 to the cognate dsDNA; however, in such case, we would not expect an increase of the helical content, but binding should be observed in the EMSA experiment.

We found that Ad30, but not its shorter form Ad26, was able to form two detectable new interaction shift bands during EMSA experiments. The shifted band detected in the absence of azoCyDdimer has a migration compatible with the monomer of Ad30, which binds to all the dsDNA sequences, inclusive to the random one (NON). Thus, this interaction was considered as unspecific. The second shift band was observed in excess of azoCyDdimer, and it corresponds to the tetra-component complex. This second band of the non-covalent tetra-component complex was observed only in the presence of CRE (′5-..ATGA cg TCAT..-3′) sequence under both illumination conditions, but not with AP1 (′5-..ATGA c TCAT..-3′), nor with mCRE (’5-..ATGAcg..-3′). The elongation of the sequence in Ad30 by insertion of the affinity enhancing QRMK sequence in before the C-terminal QGGC end, thus leading to the QRMKQGGC sequence (Ad30) instead of QGGC (Ad26), does increase the binding affinity to levels allowing the detection of the interaction of one Ad30 monomer with dsDNA containing the sequences (’5-..ATGAc..-3′). Probably the positive charge of the arginine (R) and lysine (K) groups are stabilizing effects that favor the observed interaction. In general, dynamics of the interaction may vary in a wide range depending on the truncation of the disordered dimerization domain as observed here [32]. Considering our findings, we hypothesized that the formation of a monomeric complex Ad30–azoCyDdimer and the (’5-..ATGAc..-3′) sequence might favor the subsequent tetra-component complex formation. This can be explained by the fact that when one of the sites is bound to its cognate site receptor, a second site located close-by binds cooperatively, basically because of the lower entropic cost of a (pseudo)-intramolecular interaction [35]. Considering this concept of multivalency, if the linker connecting the binding and the dimerization domains are flexible, the average distance between the sites is the main factor determining the cooperativity. In such complex, we hypothesized that the QRMKQGGC in Ad30 sequence provides the required flexibility to favor the observed tetra-covalent binding and that this region is responsible for the fuzzy behavior of the mimetic in the bound state. 

Considering that the specific migration band with CRE was also observed in both photostationary states where the E isomer is 95% and 60%, we hypothesized that the geometry of azoCyDdimer (E) is the adequate combination causing a migration band compatible with the formation of a stable tetra-component complex in the presence of ds CRE. When we evaluated the interaction of Ad30, the azoCyDdimer (E), and the CRE sequence using CD, we observed a significant increase of the helical content compatible with the formation of a tetra-component system, Ad30–azoCyDdimer (E)–Ad30–CRE, similar to those reported by the covalent dimer SS60 and other GCN4 mimetics [10,11,12,13,14,17,18]. In the case of the second photostationary state (ps) where the ps is E:Z (60:40), a moderate and lower increase of Ad30 helical content compared with the first pss E:Z (95:5) was observed, suggesting that the Z isomer does not contribute to the specific binding.

Importantly, for the interaction of Ad30–azoCyDdimer (E) and the mCRE sequence (’5-..ATGAcg..-3′), which contains only one binding site instead of the two necessary (′5-..ATGA cg TCAT..-3′) for the tetra-component formation, a much lower increase of the helical content was observed (Figure 6E). This is compatible with the interaction of one Ad30–azoCyDdimer (E) or only Ad30 with this sequence, as detected in the EMSA experiment (Figure 5C,D, lane 15). The interaction with the random sequence (NON, Figure 6F), which has no recognition motif, showed nearly no interaction, demonstrating that the interaction of the basic region with the phosphate backbone of the dsDNA has at most a minor contribution to the protein–DNA binding. Summarizing the CD experiments, the increase of the helical content upon binding is related to the bound state of Ad30–azoCyDdimer (E) with the different dsDNA as follow: CRE > AP1~ mCRE > NON. After comparison of the increase of helical content (order) of Ad30- in the presence of CRE and the two azoCyDdimer isomers, it is evident that the photo-state (E) contribute more than the (Z). It seems that dimerization interaction between the adamantane and β-cavity of the azoCyDimer occurs at the wider edge of the cyclodextrin (Figure 2A), instead of its narrow edge, which would increase the binding affinity [15]. Previously, it has been shown by molecular dynamic simulations that while the Z azoCyDdimer isomer makes a 1:1 complex with a small organic molecule with two adamantane moieties, through interaction with the narrow edge; the E azoCyDdimer isomer forms supramolecular head to tail complexes through the wider edge [16]. In the case of the mimetic of GCN4, such supramolecular aggregates are not possible because the peptide has only one binding domain. All our experiments suggested that E conformation provides the required geometry to favor the tetra-component complex, which depends on the dsDNA sequence. On the other hand, we could not discard the formation of the Ad30–azoCyDdimer (Z)–Ad30. Nevertheless, our findings indicate that the geometry of the interaction in the Z conformation is not adequate for DNA recognition.

**Figure 8:**
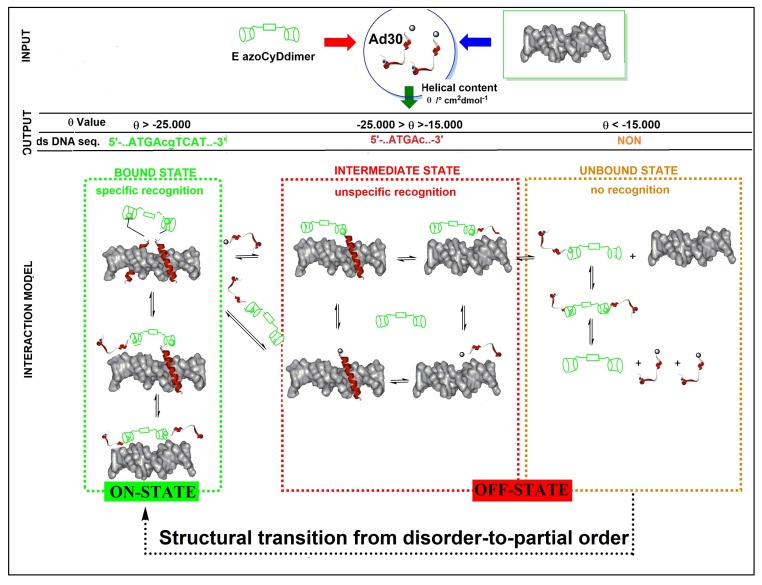
Schematic structural transitions of the Ad30 upon partner interaction with AzoCyDdimer (E) in the presence of ds CRE, representing the interacting molecules (INPUT), the obtained experimental values (OUTPUT), and the proposed interaction model. Three interaction stages are hypothesized for the system: the unbound-, the intermediate-, and the bound-state that are represented with dashed rectangles in orange, red, and green, respectively. These stages are based on the experimental helical content (θ) obtained from the individual experiments in the presence of CRE, AP1, mCRE, or NON, and the stoichiometry obtained in the EMSA experiments. In each stage, different complexes and their conformational fuzziness are shown. In the on-state *(bound state, green dashed rectangle)*, the specific binding to CRE builds up the tetra-component complex. In the bound state, the structural and dynamical continuum of fuzzy complexes is shown. The off-state is composed of many complexes, which can be unbounded to the dsDNA (*orange dashed rectangle)* or bounded but without the required sequence-specificity (*red dashed rectangle)*, thus they are referred here as the intermediate state. The structural transition forms the unbound state (disorder) to the bound state (partial order) is shown.

Finally, to confirm that the host–guest interaction between the β-CyD and the Ad moiety is a necessary requisite to recognize CRE sequence specifically, we performed a competitive assay in the presence of 1-adamantane acetic acid. Upon addition of the competitive β-CyD, the only band observed was the unspecific 1:1. A comparable result was obtained by CD upon addition of the competitive β-CyD reaching a helical content value similar to the one of the unspecific binding to mCRE. These final experiments sustain the hypothesis that the azoCyDdimer is necessary to form the homodimeric complex leading to the formation of the tetra-component system in a sequence-specific manner. We had planned to modulate the peptide–DNA interaction by the structural change promoted by the photoisomerization of the azobenzene group located as a linker between the two β-CyDs moieties in the azoCyDdimer. However, as well as for the covalent version of photomodulable GCN4 mimetic reported by Caamaño et al., it was not possible to switch the interaction effectively by changing the azobenzene conformation in situ [13]. We could not anticipate that the E isomer would be the one that favors the dimerization. Nevertheless, we have the off situation in the absence of azoCyDdimer switching the recognition to *on* above a certain azoCyDdimer (E) concentration (Figure 8). About the mechanism of interaction, we hypothesized that recognition occurs through a sequential mechanism where the monomer Ad30 probably forming a 1:1 complex with the azoCyDdimer recognizes the sequence (’5-..ATGAc..-3′) Initially with high affinity followed by the cooperative union of a second monomer promoted by the host–guest dimerization motif of azoCyDdimer in the E conformation. This second stage is stabilized by the specific interaction with CRE sequences (′5-..ATGA cg TCAT..-3′). During this interaction, different fuzzy conformers are possible that favor the specific interaction with CRE by a transition from disorder-to-partial order of the four components of the complex (Figure 8). For the others dsDNA that lack of the complete binding sequence in mCRE or only one base as a connector between the binding site in AP1, let to the formation of bi-or tri-component complexes. As described before, Tompa et al. have proposed a categorization for fuzzy complexes, as static or dynamic [1]. Considering that in the bound state, most of the Ad30 is order, it is possible to describe the interaction by the polymorphism model of static fuzziness [1]. In such model, one part of the molecule makes the contacts for the interaction whereas its dimerization domain adopts several distinct conformations, and establish the right geometry to favor dimerization and binding to the DNA with the complete recognition sequence in CRE. We hypothesized that the QRMKQGGC region of Ad30 might adopt multiple unrelated conformations to stabilize the interaction with the larger azoCyDdimer (E) instead of the compact (Z) isomer, thus favoring dimerization that contributes to CRE recognition. Probably, this structural variability limits an unfavorable decrease in entropy accompanying complex formation, which enables the combination of rapid and thermodynamically favorable binding. Truncation of this region led to no-binding as observed for Ad26, which validates our hypothesis. In conclusion, the present report is the first example of a GCN4 mimetic that forms a specific non-covalent tetra-component system with the cognate binding sequence only in the presence of an external ligand in one of two possible conformations, working as an off–on switch. Moreover, we demonstrated that chemically modified mimetics of GCN4 are suitable minimalist models to investigate conformational fuzziness in protein–DNA interactions, opening the opportunity to investigate biomolecular interaction by implementing fuzzy logic sets as proposed by Gentili [36].

## 4. Materials and Methods

### 4.1. Peptide Synthesis

Disulfide dimers SS52 and SS60 were synthesized from commercial peptides SH26 and SH30, to have reference standards to study their interactions with DNA [15]. Both products were purified by semi-preparative reverse phase HPLC (Waters, Milford, MA, USA) and then lyophilized. The characterization was carried out by MALDI–TOF (Bruker Daltonik, Bremen, Germany), SS52: (M + H) calc. C_234_H_414_N_98_O_74_S_2_ 5786, found: 5788.5 (43%yield). SS60: (M + H)^+^ calc. C_278_H_498_N_116_O_80_S_4_ 6873.95, found: 6875.36 (50% yield).

For the synthesis of Ad26 [15] and Ad30, on both deoxygenated solutions of SH26 (1.9 mg, 6.2 × 10^−4^ mmol) and SH30 (2.0 mg, 5.82 × 10^−4^ mmol) in potassium phosphate buffer (150 μL, 100 mM, pH = 8.0) and CH_3_CN 50 μL were added 4 equiv of bromo acetyldamantane in CH_3_CN (0.79 mg, 9 μL). The mixtures were stirred at room temperature for 3 h under N_2_ and checked by RP-LC–MS (Agilent 1100, Santa Clara, CA, USA),. The new compounds were purified by semi-preparative RP-HPLC and then lyophilized, identified by mass spectrometry as the alkylated peptides. Once purified and lyophilized, Ad26 (59%) and Ad30 were obtained in 59% and 66% yield, respectively. MALDI–TOF for Ad26, C_130_H_229_N_49_O_37_S_1_: calc. (M + H)^+^ 3101.36, found 3102.81; and for Ad30, C_152_H_272_N_58_O_42_S_2_: calc. (M + H)^+^ 3646.04, found 3647.08. 

### 4.2. Synthesis and Characterization of azoCyDdimer

4,4′-bis (carboxy) azobenzene (0.036 g, 0.132 mmol) was dissolved in dry DMF (2 mL), HATU in DMF was added (0.10 g, 0.317 mmol) and 0.183 mL DIPEA (0.136 g, 1.056 mmol), was stirred for 5 min at room temperature, and to this solution was added β-CyD-NH2 (0.300 g, 0.264 mmol) dissolved in 2 mL of DMF, the resulting mixture was stirred for 3 h under nitrogen atmosphere. The mixture was poured over a container with ice-cold acetone and a precipitate appeared. The solid was separated by filtration and then dissolved in DMF to be purified by silica gel chromatography column. (CH_3_CN–H_2_O–NH_4_OH 14:0:0.5/4:10:0.5). ^1^H-NMR (500 MHz (Avance II 500, Bruker, Germany), DMSO-d6) δ (ppm): 3.3–4.33 (m, 42H ov 2 H_2_O), 4.33–4.56 (br.s, 12H), 4.96 (br.s, 14H), 4.82 (br.s, 7H), 5.7–5.82 (m, OH, 27H), 7.98 (4 H, d, *J* = 8.6 Hz), 8.04 (4 H, d, *J* = 8.6 Hz), 8.50 (2 H, br. s). ^13^C-NMR (125.75 MHz, DMSO-d_6_) δ (ppm): 41.8 (CH_2_), 59.7 (CH_2_), 71.9 (CH), 72.3 (CH), 73.0 (CH), 81.3 (CH), 81.9 (CH), 84.1 (CH), 101.64 (CH), 101.9 (CH), 122.2 (CH), 128.4 (CH), 137.4 (C), 153.7 (C), 166.2 (C). MALDI–TOF (M + H)^+^: calcd. for C_98_H_148_N_4_O_70_ 2500.8, (M + Na)^+^: calc. 2523.58, found 2523.53, (M + K)^+^: calc. 2539.98, found 2539.96.

#### H-NMR Photoisomerization Experiment

Four independent photoisomerization experiments were performed using different initial concentrations of azoCyDdimer: 15 mM, 8 mM, 2.28 mM, and 0.5 mM. Dilutions were made from a 15 mM stock solution with an isomer ratio of 95:5 (E:Z). The stock solution and the dilutions were irradiated at 360 nm for 20 min, and then the corresponding ^1^HRMN spectra were acquired (AMX 300, Bruker, Germany), protecting the solution from the visible light. Switching experiments were performed with an 8 W mercury arc lamp with filter of 360 nm from Pleuger, Antwerp, Brussels.

### 4.3. Annealing of dsDNA

Oligonucleotides were purchased from Thermo Fisher Scientific GmbH on a 0.2 mmol scale as freeze-dried solids. After solving in H_2_O milliQ, their concentrations were measured by ultraviolet absorption at 260 nm with a BioRad SmartSpec Plus Spectrophotometer. Absorbance was measured twice, and concentrations were calculated applying Lambert–Beer’s equation. The molar extinction coefficients of single-strand oligonucleotides were calculated by using the following formula ε(260 nm) = {(8.8 × #T) + (7.3 × #C) + (11.7 × #G) + (15.4 × #A)} × 0.9 × 10^3^ M^−1^ cm^−1^, where #A, #T, #C, #G stand for the number of each type of bases in the DNA strand. Oligonucleotides were annealed by heating an equimolar mixture of the two complementary single-strand DNAs to 90 °C for 2 min and then cooling slowly to room temperature (12 h).

### 4.4. Electrophoretic Shift binding Assays

EMSA was performed with a BioRad Mini Protean gel system, powered by an electrophoresis power supplies PowerPac Basic model, maximum power 150 V, frequency 50.60 Hz at 140 V (constant V). The binding reactions were performed over 30 min in a binding mixture (20 or 40 μL) containing 18 mm tris(hydroxymethyl)aminomethane (Tris; pH 7.5), 90 mm KCl, 1.8 mm MgCl_2_, 1.8 mm EDTA, 9%glycerol, 0.11 mgmL^−1^ bovine serum albumin (BSA), and 2.2% NP-40 (nonidet-P40). Products were resolved by PAGE by using a 10% nondenaturing polyacrylamide gel and 0.5XTBE buffer solution (44.5 mm Tris, 44.5 mm boric acid,1 mm EDTA, pH 8) and analyzed by staining with SyBrGold (Molecular Probes: 5 mL in 50 mL of 1XTBE) for 10 min and visualized with fluorescence. Ad26 working concentration was 200 nM, 50 nM of DNA, and for azoCyDdimer was used from 0 to 100 equivalents of the E and Z dimer, respectively, was selected (stock solution 0.74 mM in H_2_O, ratio E:Z determined by RP-HPLC, Supplementary Materials Figure S5). The order of addition was azoCyDdimer, Ad26 (pre-incubation for 10 min at 4 °C), and then the corresponding DNA. Duplicates of independent experiments were performed. For the competitive binding assay, we added 200 equiv of 1-adamantane acetic acid after the addition of azoCyDdimer.

### 4.5. Circular Dichroism Spectroscopy

CD experiments were performed on a Jasco spectrometer (Jasco 715, Tokyo, Japan) with 1 mm path-length cell [17]. Samples in 10 mM phosphate buffer (pH 7.0) and 100 mM NaCl contained 10 or 5 µM of peptide and 5 µM oligonucleotides (double-stranded) in the absence or presence of 1 equiv of azoCyDdimer at 4 °C (stock solution 0.74 mM in H_2_O, ratio E:Z determined by RP-HPLC, see Supplementary Materials Figure S5). Every sample was incubated for 5 min before registering. Duplicates of independent experiments were performed. The CD spectra of the peptides (when measured in the presence of DNA) were calculated as the difference between the spectrum of the peptide/DNA mixture and the measured spectrum of the respective dsDNA oligonucleotide. For the competitive binding assay, we added 200 equiv of 1-adamantane acetic acid after the addition of azoCyDdimer. Smoothing of the signals was performed by using the software KaleidaGraph.

## Supplementary Materials

The following are available online at https://www.mdpi.com/1420-3049/24/13/2508/s1, Figure S1: ^1^H-NMR of the azoCyDdimer, Figure S2: 2D-NMR-HSQC of azoCyDdimer, Figure S3: 2D-NMR-HMBC of azoCyDdimer, Figure S4: UV–Vis Spectra of azoCyDdimer depending on illumination conditions; Figure S5 RP-LC–MS of azoCyDdimer depending on illumination conditions. Tables S1 and S2: ^1^H-NMR and ^13^C-NMR chemical shifts of AzoCyDdimer (E).
