# Supplementary material for: Sequence-Specific DNA Binding by Noncovalent Peptide–Azocyclodextrin Dimer Complex as a Suitable Model for Conformational Fuzziness
Source: Molecules. 2019 Jul 9;24(13):2508. doi: 10.3390/molecules24132508 (PMC6650922; doi:10.3390/molecules24132508)
Supplement: Supplementary file 1 [file molecules-24-02508-s001.pdf]

Supplementary Information

Sequence-Specific DNA Binding by Noncovalent Peptide–Azocyclodextrin Dimer Complex as a Suitable Model for Conformational Fuzziness.

Zulma B. Quirolo [1,2,3,†], M. Alejandra Sequeira [1], José C. Martins [2] and Verónica I. Dodero [1,3,†,*]

[1] Depto. Química- Universidad Nacional del Sur, Bahía Blanca 8000 Argentina
[2] NMR and Structure Analysis, Department of Organic and Macromolecular Chemistry, Ghent University, Ghent 9000, Belgium
[3] Faculty of Chemistry, Bielefeld University, Universitätsstr. 25, 33615 Bielefeld, Germany
* Correspondence: veronica.dodero@uni-bielefeld.de; Tel.: +49-(0)521-106-2046; Fax: +49-(0)521-106-156963
[†] Current affiliation: IFISUR – Depto. Química - Universidad Nacional del Sur, Bahía Blanca 8000, Argentina

Table of Contents.

1. ^{1}H and ^{13}C-NMR spectra of the azoCyDdimer **S2**

2. UV-Visible spectra and HPLC of the azoCyDdimer **S4**

3. HPLC and MALDI-Tof of peptides derivatives **Ad30** and **Ad26** **S5**

1. ^{1}H and ^{13}C-NMR spectra of the azoCyDimer

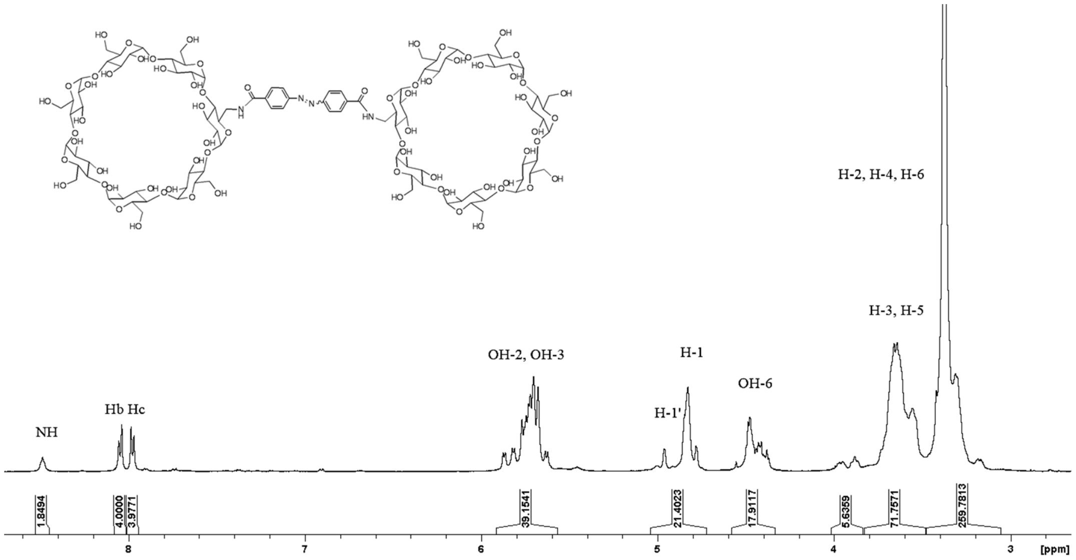

Figure S1. ^{1}H-NMR of the dimer 500MHz. DMSO-d_6 a 25 °C.

Through an HSQC experiment, the determination of the ^{1}H-^{13}C correlations through a bond was made and allowed CH_2 differentiation of CH. The CH_2 have reverse phase respect to the CH signals. In this type of experiments, quaternary carbons cannot be determined. The analysis was completed with HMBC, which allows establishing the correlations ^{1}H and ^{13}C at long distance (two or three bonds).

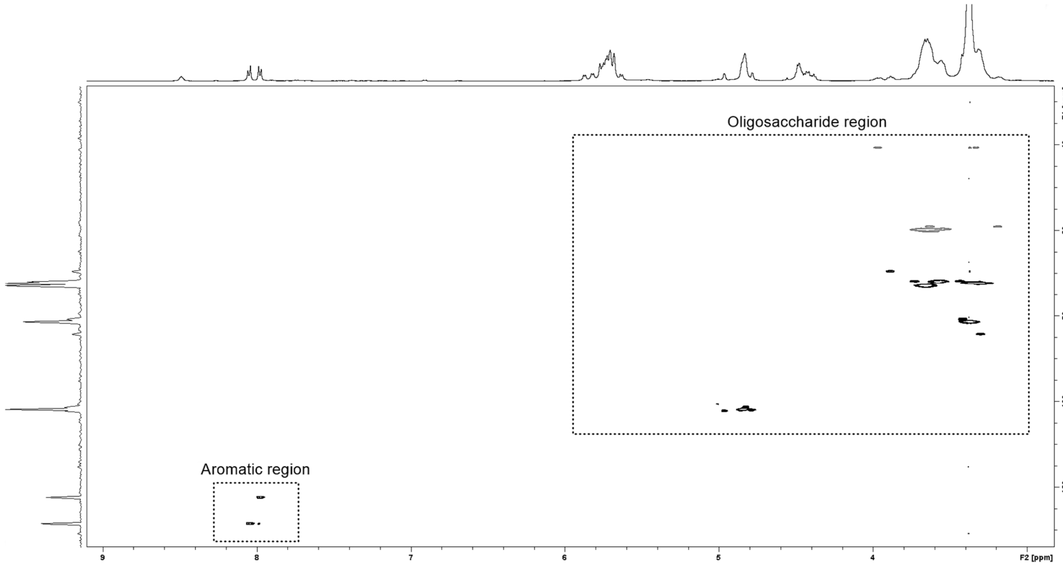

Figure S2. 2D NMR - HSQC -500MHz in DMSO-d6 a 25 °C.

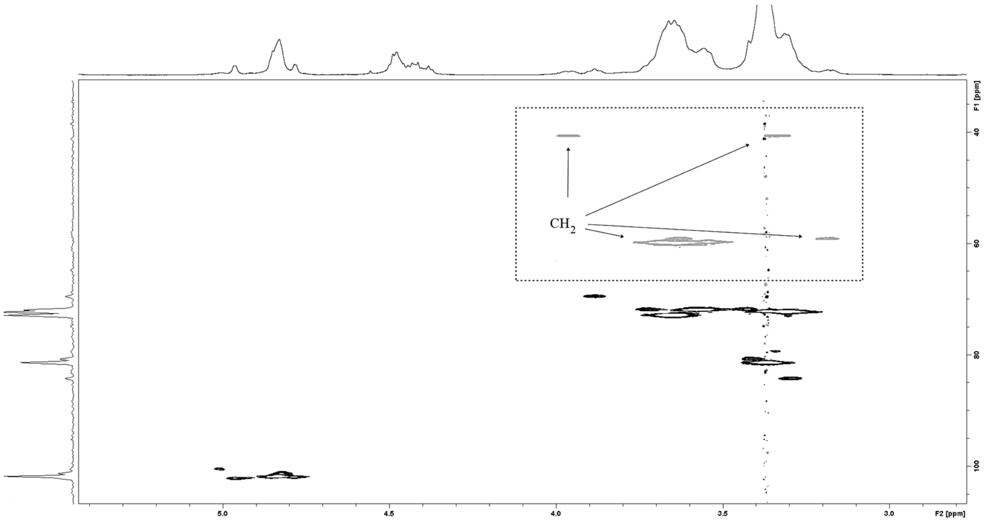

Figure S3. 2D NMR- HMBC -500MHz in DMSO-d6 a 25 °C (Oligosaccharide region).

Table S1. Table of chemical shifts of AzoCyDdimer (E).

CyD[1]	H1	H1′	H6	H6′	H6″	H5	H3	H4	H2
^{1}H ppm	4.96	4.83	3.65	3.96	3.33	3.31	3.65	3.37<br>3.20	3.29
Area	14 H, br.s		84 H, ov[2].80 H_2O (H6, H6’, H’’6, H5, H4, H2)						
^{13}C ppm	101.95	101.64	59.7	40.54	40.53	72.17	72.77	81.29<br>84.05	81.9

[1] NMR signals from the cyclodextrin of the dimer. [2] The signal overlaps with that of the water

Table S2. Table of chemical shifts of AzoCyDdimer (E).

Conector[1]	N-Ha	Hc	Hb	C-Ar2	C-Ar1	C-O
^{1}H ppm	8.50	7.98	8.04			
Area	2 H, br. s	4 H, d<br>(*Jo* = 8.6 Hz)	4 H, d<br>(*Jo* = 8.6 Hz)			
^{13}C ppm		122.22	128.35	137.4	153.7	166.2

[1] NMR signals from the azobenzene region of the dimer

2. UV-Visible spectra and HPLC of the azoCyDdimer

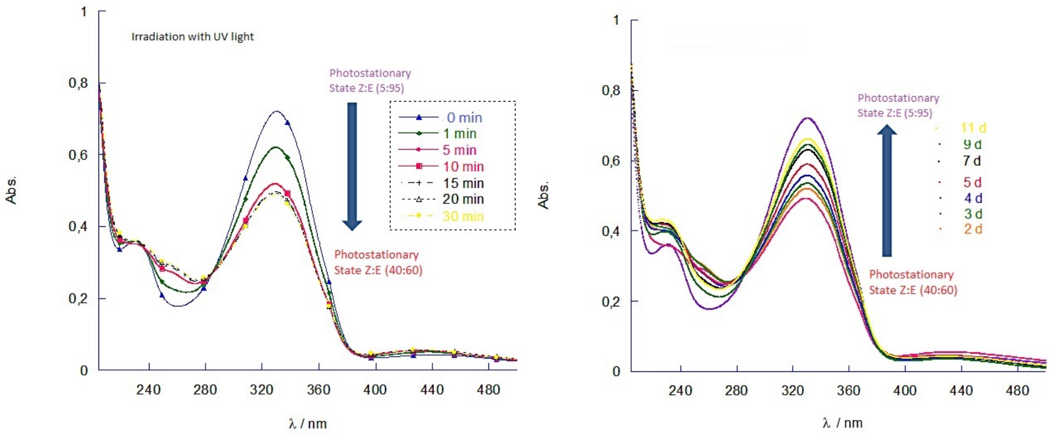

Figure S4. AzoCyDdimer solution in H_2O (0.037 mM) after irradiation at 360 nm at different times. b) Overlapped spectra of the photostationary mixture reversion in the dark after 11 days.

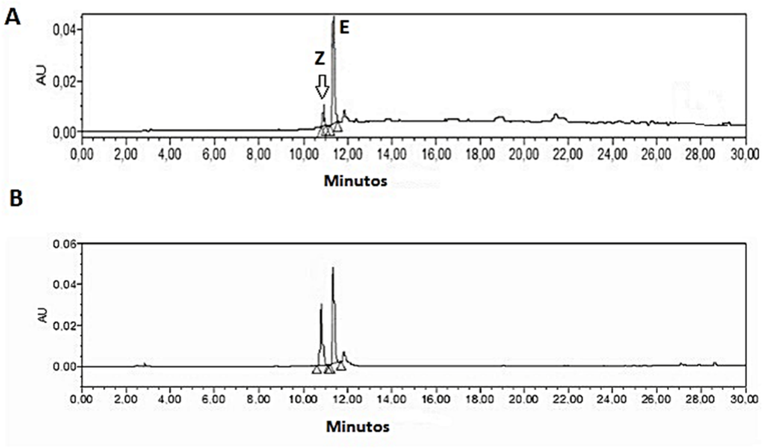

Figure S5. A) RP-LC-MS of azoCyDdimer solution in H_2O. (0.74 mM) B) Dimer solution after been irradiated at 360 nm for 20 min. (A: H_2O; B: ACN; Gradient: 5-95%B in 30 min. Detection at 280 nm).

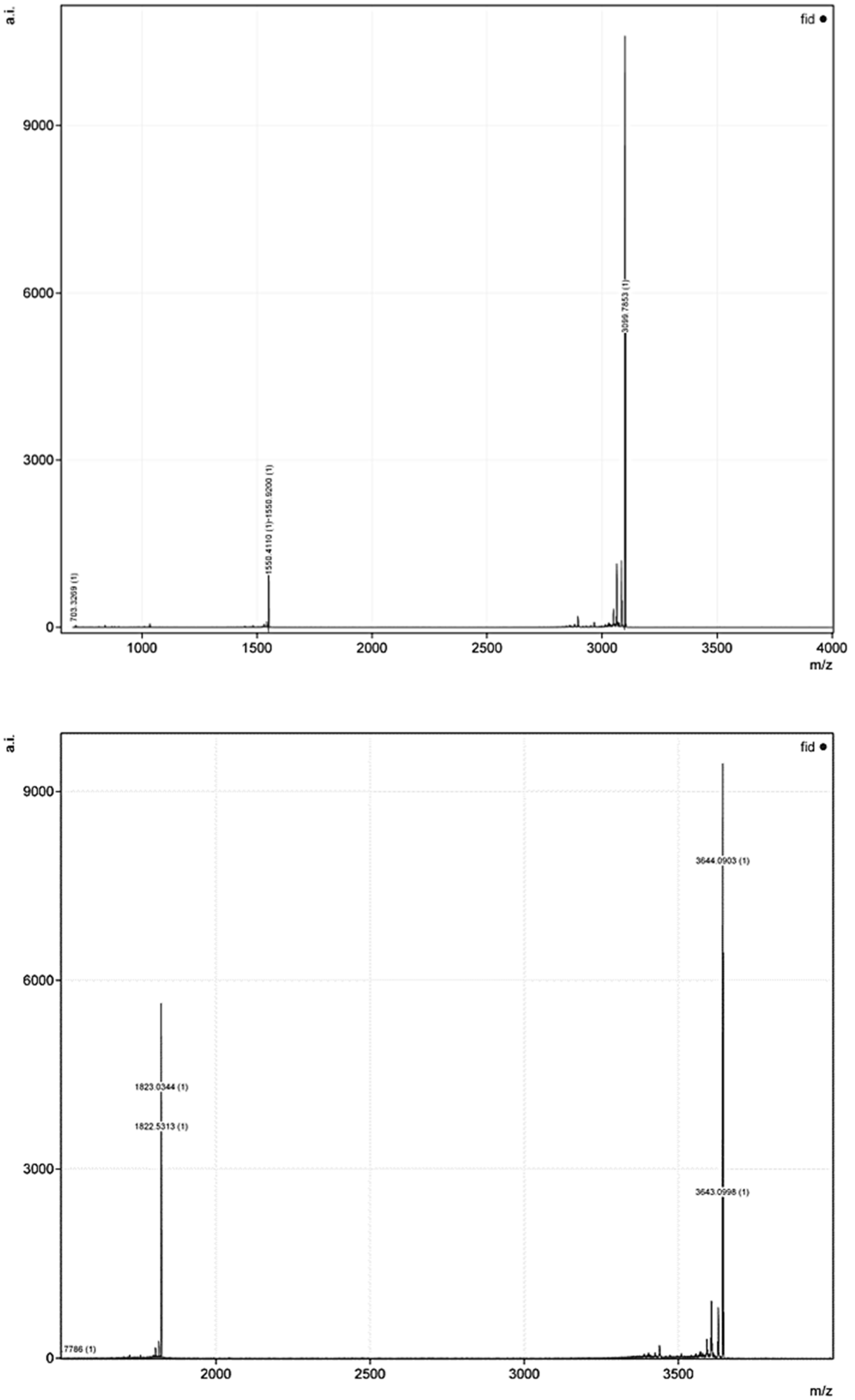

Figure S6. MALDI-TOF of derivatives Ad26 and Ad30
